# Prognostic significance of tumor-associated macrophages in breast cancer: a meta-analysis of the literature

**DOI:** 10.18632/oncotarget.15736

**Published:** 2017-02-25

**Authors:** Xixi Zhao, Jingkun Qu, Yuchen Sun, Jizhao Wang, Xu Liu, Feidi Wang, Hong Zhang, Wen Wang, Xingcong Ma, Xiaoyan Gao, Shuqun Zhang

**Affiliations:** ^1^ Department of Oncology, The Second Affiliated Hospital of Xi'an Jiaotong University, Xi'an, Shaanxi, P.R. China; ^2^ The Second Department of Thoracic Surgery, The First Affiliated Hospital of Xi'an Jiaotong University, Xi'an, Shaanxi, P.R. China; ^3^ The Department of Radiation Oncology, The First Affiliated Hospital of Xi’an Jiao Tong University, Xi’an, Shaanxi, P.R. China

**Keywords:** breast cancer, tumor associated macrophages, prognosis, meta-analysis

## Abstract

**Purpose:**

Tumor associated macrophages (TAMs) are important prognostic factors and have been proved to be associated with the invasion and migration of various cancer. However, the relationship between TAMs and breast cancer outcomes remains unclear.

**Experimental Design:**

Sixteen studies with a total of 4,541 breast cancer patients were included in this meta-analysis. Correlation of TAMs with overall survival (OS), disease-free survival(DFS), relapse-free survival (RFS), breast cancer special survival (BCSS) and clinicopathological features were analyzed. Survival data and clinicopathological value were integrated by analyzing hazard ratio(HR) and odds ratio(OR) separately and using Fixed-effect or Random-effect model according to heterogeneity. All statistical tests were two-sided.

**Results:**

OS and DFS were correlated with high density of TAMs with HR= 1.504(1.200, 1.884)/ 2.228(1.716, 2.892) respectively. And subgroup analysis of location and biomarker in OS and DFS group showed prognosis was associated with TAMs distribution and biomarker selection. Besides, TAMs high infiltration was significantly related to age, size, histologic grade, ER/PR status, basal phenotype and vascular invasion.

**Conclusion:**

High density of TAMs was associated with poor survival rates of breast cancer. TAMs in stroma are associated with worse outcome than that in nest and using CD68 as a biomarker for TAMs to evaluate the risk is better than CD163 or CD206 alone. Moreover, high infiltration of TAMs was significantly associated with negative hormone receptor status and malignant phenotype. TAMs infiltration can serve as a novel prognostic factor in breast cancer patients.

## INTRODUCTION

Breast cancer is the most frequent cancer in female. In recent years, with the trend of younger age and increasing morbidity, it threatens the health of women seriously, but the mechanism isn't clear [1, 2]. Breast cancer has obvious heterogeneity on molecular phenotype, tissue pathology and clinical characteristics. Nowadays, the focus of treatment strategy is using chemotherapy to induce tumor cell apoptosis, resistance to hormone receptor and targeted therapy. Meanwhile, growing evidence has demonstrated the important role of tumor microenvironment in the development of cancer and the treatment aim at interfering the microenvironment and metabolism stimulation has gained clinical utility [3–5]

Macrophage is a main kind of immune cell infiltrating to tumor microenvironment and it can change its phenotype according to the signal stimulation of microenvironment. TAMs are generally characterized by the expression of cell surface marker CD68 [6]. Macrophages can be divided into classically activated M1 and selectively activated M2 [7, 8]. M1 macrophages are induced by TLR (toll like receptor) and IFN-γ, expressing high level of CD86, CD40 and PD-L1 and play the role of pro-inflammatory and anti-tumor response [9]. While M2 is totally different, they are characterized by the expression of CD163, CD206, CD204 and arginase-1, secreting IL-10 and TGF-β, expressing VEGF (vascular endothelial growth factor) and promoting both the activation of tumor related regulatory T cells and matrix formation, all of which promote tumor progression [10–12].

Plentiful studies have confirmed that TAMs are associated with poor outcome of human cancer, such as hepatoma, gastric cancer, lung cancer and so on [13–16]. However, other studies have opposite reports [17]. Studies reveal that macrophage subtype, location, density are all in correlation with cancer survival [18, 19]. For breast cancer, studies have shown that density of TAMs is related to hormone receptor status, stage, lymph node metastasis and poor prognosis [2, 18–21]. However, further studies are needed to clarify the influence of TAMs including the role of intratumoral distribution and surface marker selection. Therefore, we conducted a detailed meta-analysis summarizing the related evidence to evaluate the prognostic value of TAMs in human breast cancer.

## RESULTS

### Search results and study characteristics

**Figure 1:**
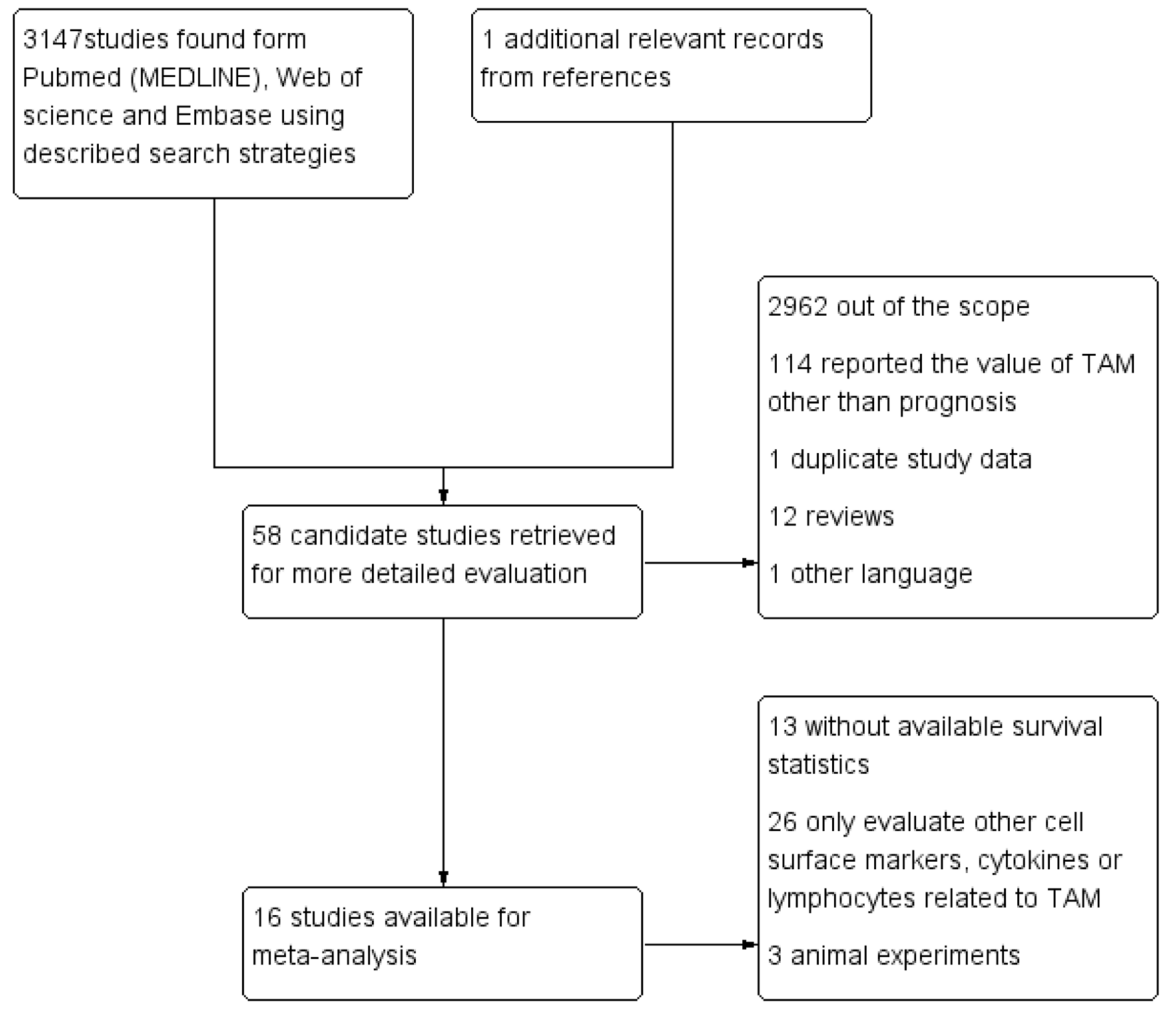
Selection of studies Flow chart showed the selection process of the included studies

Literature retrieval yield 3148 records and we selected 58 candidate studies (Figure 1). By further screening, 42 articles are excluded because of lacking survival data and canine tissue samples. Finally, 16 citations with valid survival data were included.

The characteristics of included study containing OS, DFS, RFS, BCSS data were listed in Table 1. There are 4541 patients in total of included studies. And the sample size for OS, DFS, RFS, BCSS was 1699, 916, 1593, 1634 separately. All the studies used IHC staining in formalin-fixed paraffin-embedded tissue blocks to evaluate TAMs biomarker expression. When evaluating TAM density, 50% studies performed blinded and independent reading. Cut-off value for definition of TAMs density low versus high could be retrieved from 15 articles (Table 1).

**Table 1:** Characteristics of the eligible studies

Ref	Patient No.	Age median (range)	Follow-up median (range)	Cutoff value	Macrophage density	Tissue distribution	Analysis	Marker	Company	Stage	NOS Score	Result
Koru-Sengul,T. (2016)	150	54.9±12.4	116 (2-326)	1+=1–150 cells/mm^2^ 2+=151–300 cells/mm^2^ 3+=> 300 cells/mm^2^ CD163/CD206 2+/3+ vs 1+ CD40 1+/2+ vs 0	cells/mm^2^	tumor nest and stroma	blind and independent	CD163 CD206 CD40	CD163/206/40 R&D,Minneapolis	I-IV	8	OS
Yang, J. (2015)	100	55 (28-80)	60	>61.14±23.76 <61.14±23.76	high-power field(HFP)	tumor nest and stroma /peritumoral stroma	no	CD68	DAKO, Carpinteria, CA	I-IV	6	OS
Sousa, S. (2015)	562	52.5 (26-65)	35 (25.5–43.6)	>369;≤369	cells/mm^2^	tumor mest and stroma	double- blinded fashion	CD68 CD163	CD68(Abcam,CambridgeUK);CD163(Novocastra, New-castle,UK)	I-IV	8	RFS
Gwak,J.M. (2015)	372	50.96 (26-87)	92.4 (1.2-127.2)	intratumoral:>24.2;≤24.2 stromal:>35.3;≤35.3	high-power field(HPF)	tumor nest vs stroma	no	CD68	Dako, Carpinteria, CA	I-III	7	DFS
Satu Tiainen (2015)	278	59 (32-86)	75.6 (4.8-133.2)	CD163:>26;≤26 CD68:>34;≤34	high-power field(HPF)	tumor nest and stroma	blind and independent	CD163 CD68	Thermo Scientific	I-III	8	OS
Yuan,Z.Y. (2014)	287	NA	89 (4–181)	>16;≤16	high-power field(HPF)	stroma	no	CD68	Thermo Fisher Scientific, Waltham, MA, USA	I-III	7	OS/DFS
Zhang,Y. (2013)	172	49 (29-73)	60	≥26 /tissue cores <26 /tissue cores	high-power field(HPF)	stroma	blind	CD68	Abcam, USA	I-IV	7	OS/DFS
Carrio,R. (2013)	29	59 (32-87)	138 (24-292)	≥5 TAMs/slide; <5 TAMs/slide	high-power field(HPF)	tumor nest	blind	CD68	Carpinteria, CA, USA	I	7	OS
Campbell,M.J (2013)	102	NA	100	>24;≤24	high-power field(HPF)	tumor nest and stroma	no	CD68+ /PCNA+	Dako, Cambridgeshire, UK	I-III	7	RFS
Mohammed,Z.M. (2012)	468	NA	165	≥3%; <3%	high-power field(HPF)	tumor nest and stroma	blind and independent	CD68	Dako, Glostrup, Denmark	I-III	8	RFS
Medrek, C. (2012)	144	65 (34-97)	78.6 (3.96-90.6)	absent/sparse (0-2) dense (3)	high-power field (HPF)	tumor nest vs stroma	no	CD68 CD163	CD163 Novocastra CD68 DAKO	I-III	8	OS/RFS/BCSS
Mahmoud,S.M. (2012)	1322	<70	127 (1-192)	intratumoral:>6;≤6 stromal:>17;≤17	high-power field(HPF)	tumor nest and stroma	blind	CD68	Dako, Glostrup,Denmark	I-III	6	BCSS
Campbell,M.J (2011)	216	55.75	108	>5;≤5	high-power field(HPF)	tumor nest and stroma	no	CD68+ /PCNA+	Dako, Cambridgeshire, UK	I-III	8	OS/RFS
Mukhtar,R.A. (2011)	70	50 (30-74)	87.6 (6–156)	>38.5;≤38.5	high-power field(HPF)	tumor nest and stroma	blind and independent	CD68+ /PCNA+	Dako, Cambridgeshire, UK	I-III	8	OS
Murri,A.M. (2008)	168	>50 (81%)	72	NA	NA	tumor and surrounding stroma	blind and independent	CD68	Dako, Cambridgeshire, UK	I-III	7	BCSS /OS
Leek,R.D. (1996)	101	NA	60	>12;≤12	high-power field(HPF)	tumor nest and stroma	no	CD68	Dako, Cambridgeshire, UK	NA	6	RFS

Abbreviations: NA: Not available; OS: overall survival; DFS: disease-free survival; RFS: relapse-free survival; BCSS: breast cancer specific survival; NOS: Newcastle-Ottawa Scale checklist.

### TAMs definition and density

The introduction of the antibodies and definition method of TAMs of included studies were shown in Table 1. 15 studies used CD68, 4 studies used CD163 and only one study used CD206 and CD40. Of all the studies using CD68, 18.75% using CD68+/PCNA+.

### Data synthesis: clinicopathological features

Our results showed that high TAMs infiltration were significantly correlated to age (OR = 1.211 (1.031, 1.422), fixed-effect model), size (OR = 0.73 (0.616, 0.865), fixed-effect model), histologic grade (OR = 0.344 (0.257, 0.459), random-effect model), ER status (OR = 2.760 (1.808, 4.213), random-effect model), PR status (OR = 2.188 (1.825, 2.623), fixed-effect model), basal phenotype (OR = 0.436 (0.346, 0.550), fixed-effect model), vascular invasion (OR = 0.623 (0.509, 0.763), fixed-effect model). On the contrary, high TAMs infiltration was not found to be associated with lymph node status (OR = 0.843 (0.646, 1.099), random-effect model), HER-2 status (OR = 0.807 (0.459, 1.418), random-effect model). These results indicated that breast cancer with high TAMs infiltration exhibited aggressive biological behaviors (Table 2).

**Table 2:** Meta-analysis for the association of increased total TAMs expression and clinicopathological features of breast cancer patients

Clinicopathological features	No.of studies	No.of patients	Model	OR(95% CI)	P-Value	Heterogeneity		
						I^2^	I^2^(%)	P-Value
Age(≤50 vs. >50)	6	2708	Fixed	1.211(1.031,1.422)	0.02	3.45	0	0.63
Size(≤2cm vs. >2cm)	6	2698	Fixed	0.73(0.616,0.865)	0	7.41	32.5	0.192
Histologic grade(G1-2 vs. G3)	9	3141	Random	0.344(0.257,0.459)	0	20.96	61.8	0.007
lymph node status(N0 vs. N1-3)	6	2904	Random	0.843(0.646,1.099)	0.206	13.71	48.9	0.057
ER status(Negative vs. Positive)	7	2788	Random	2.760(1.808,4.213)	0	31.74	77.9	0
PR status(Negative vs. Positive)	5	2159	Fixed	2.188(1.825,2.623)	0	4.59	0	0.468
HER-2(Negative vs. Positive)	7	2558	Random	0.807(0.459,1.418)	0.456	45.37	84.6	0
Basal phenotype(Negative vs. Positive)	6	2268	Fixed	0.436(0.346,0.550)	0	0.97	0	0.915
Vascular invasion(Absent vs. Present)	3	1869	Fixed	0.623(0.509,0.763)	0	3.14	36.4	0.208

### Data synthesis: overall survival

**Figure 2:**
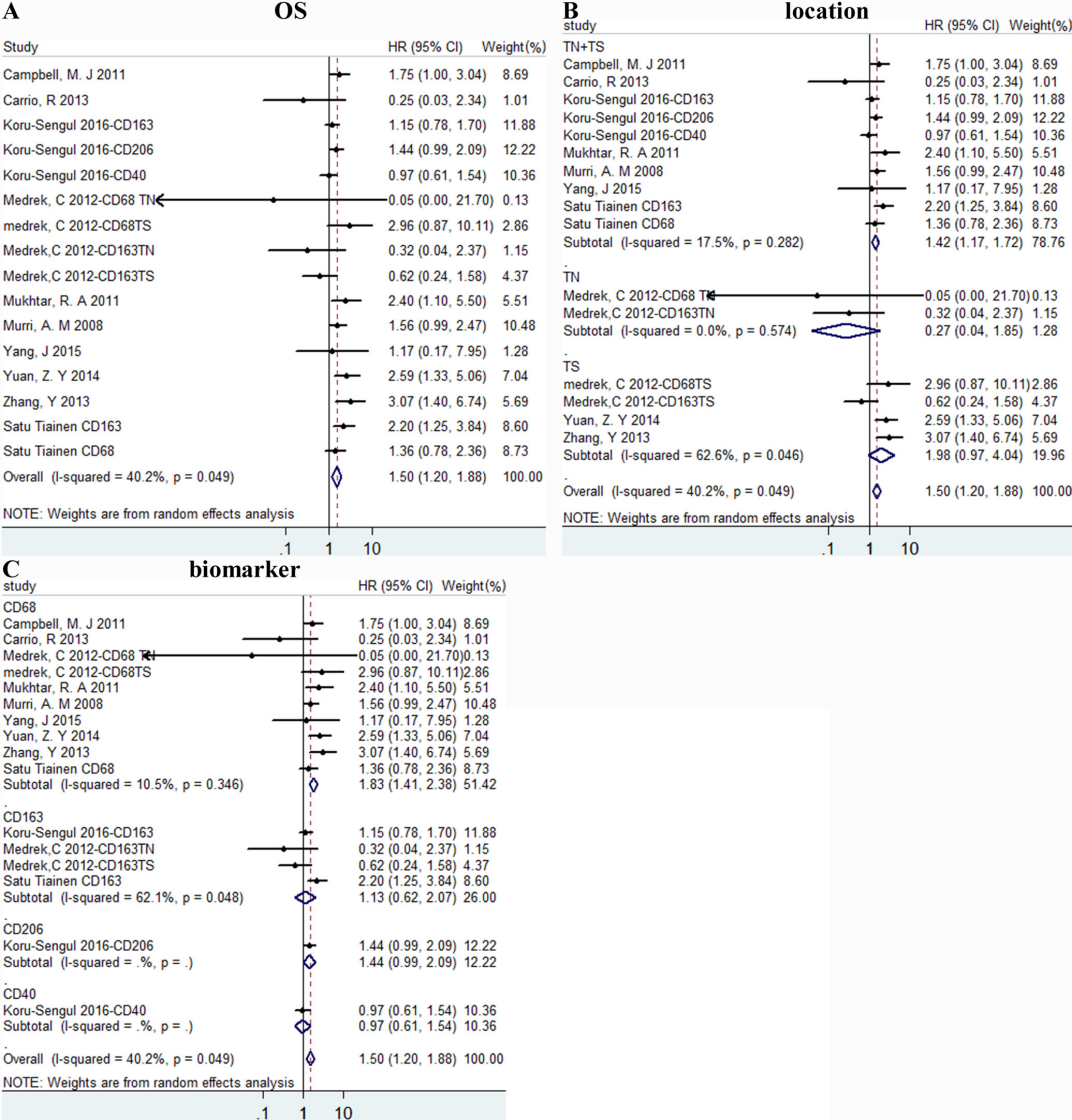
The forest plots of HRs for OS Forest plots and meta-analysis of studies evaluating HRs of high TAM counts as compared to low counts. Survival data were reported as OS **A**., as well as subgroup analysis of location **B**. and biomarker **C**. among included studies. TN: tumor nest; TS: tumor stroma.

High density of TAMs was associated with poor OS, HR = 1.504 (1.200, 1.884), I^2^ = 40.2% (Figure 2A). Besides, the results of subgroup analysis according to location were 1.42 (1.17, 1.72), 0.27 (0.04, 1.85), 1.98 (0.97, 4.04) of TN+TS (tumor nest and stroma), TN (tumor nest) and TS (tumor stroma) group separately (Figure 2B). According to biomarker, HR = 1.83 (1.41, 2.38)/1.13 (0.62, 2.07)/1.44 (0.99, 2.09)/0.97 (0.61, 1.54) of CD68, CD163, CD206, CD40 separately(Figure 2C).

### Data synthesis: disease free survival

**Figure 3:**
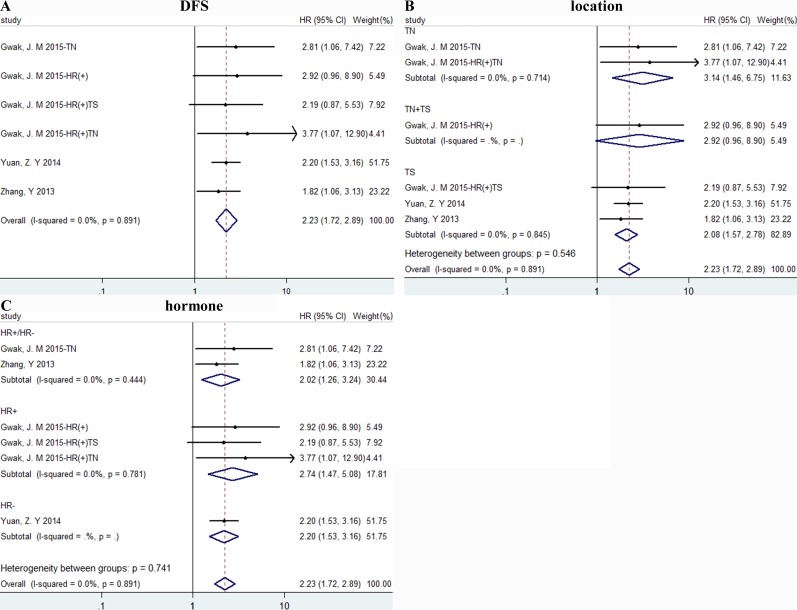
The forest plots of HRs for DFS Forest plots and meta-analysis of studies evaluating HRs of high TAM counts as compared to low counts. Survival data were reported as DFS **A**., as well as subgroup analysis of the location **B**. and hormone status **C**. of included studies. TN: tumor nest; TS: tumor stroma.

High density of TAMs was related to poor DFS, HR = 2.228(1.716, 2.892), I^2^ = 0% (Figure 3A). And results were associated with TN (tumor nest) and TS (tumor stroma) with HR = 3.14 (1.46, 6.75)/2.08 (1.57, 2.78) separately, I^2^ = 0% (Figure 3B). And we also conducted a subgroup meta-analysis according to hormone status and results in all subgroups showed obvious significance (Figure 3C).

### Data synthesis: relapse free survival

**Figure 4:**
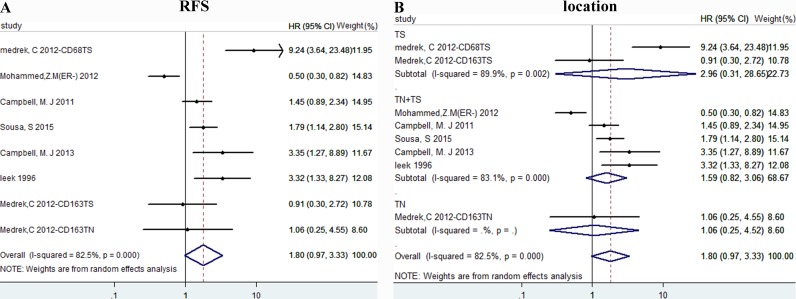
The forest plots of HRs for RFS Forest plots and meta-analysis of studies evaluating HRs of high TAM counts as compared to low counts. Survival data were reported as RFS **A**., as well as subgroup analysis of the location **B**. Abbreviations: TN: tumor nest; TS: tumor stroma.

We didn't find relationship between TAMs density and RFS, with HR = 1.799 (0.972, 3.330), I^2^ = 82.5% (Figure 4A). Then subgroup analysis was conducted to find the origin of heterogeneity. According to location of TAMs, we divided our studies into three groups: TS, TN+TS, TN. However, because of different biomarker, hormone status within these studies, it's hard to eliminate heterogeneity (Figure 4B).

### Data synthesis: breast cancer special survival

**Figure 5:**
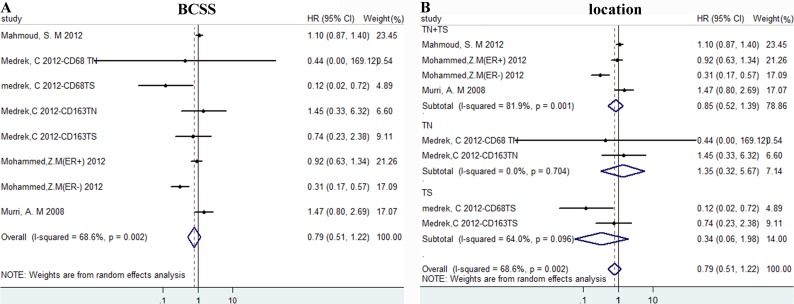
The forest plots of HRs for BCSS Forest plots and meta-analysis of studies evaluating HRs of high TAM counts as compared to low counts. Survival data were reported as BCSS **A**., as well as subgroup analysis of the location **B**. Abbreviations: TN: tumor nest; TS: tumor stroma.

Results of high density of TAMs *versus* low showed no difference for BCSS, HR = 0.786 (0.505, 1.222), I^2^ = 68.6% (Figure 5A). Then we conducted subgroup analysis according to location and there was still no difference(Figure 5B).

### Publication bias

**Figure 6:**
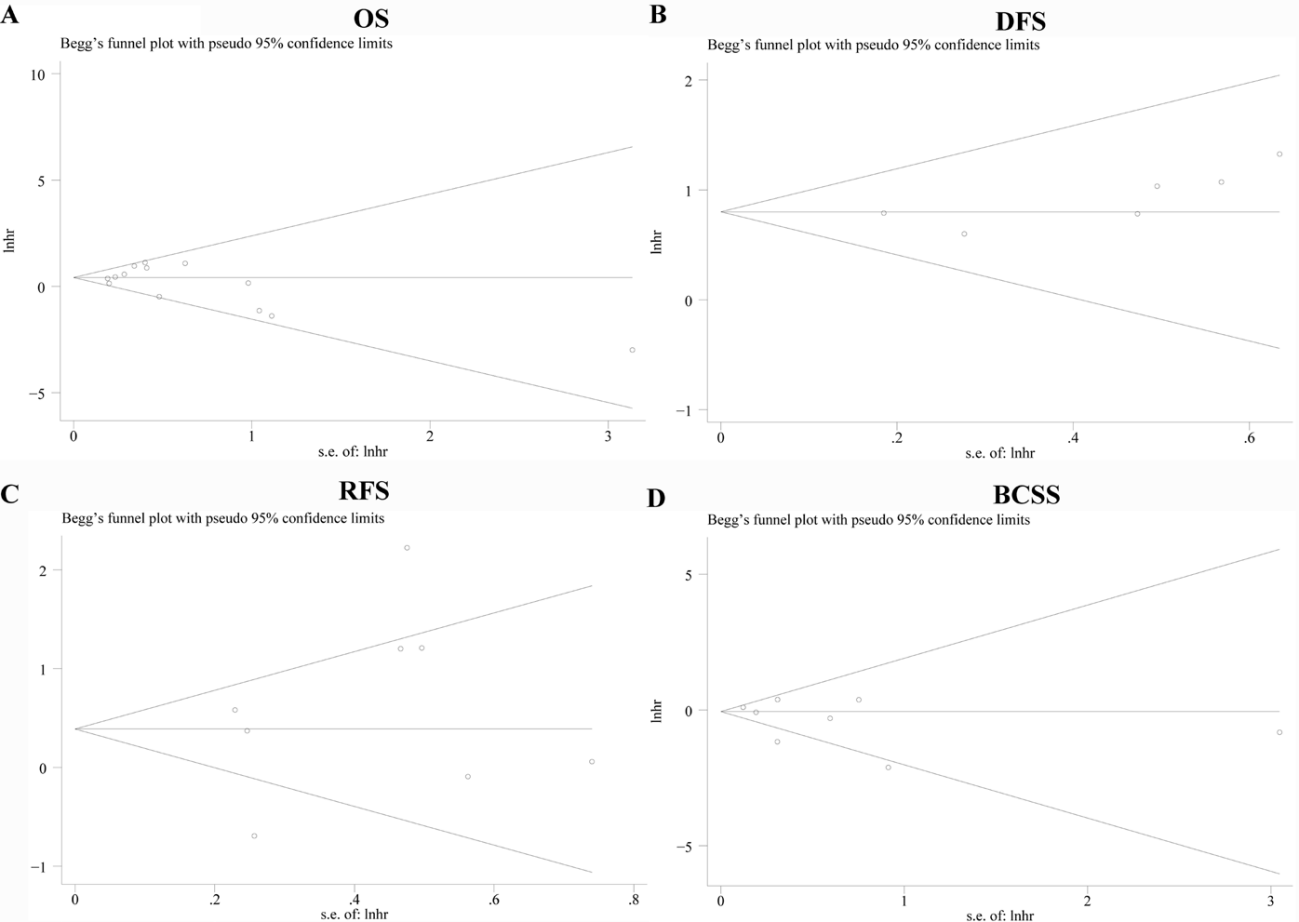
Funnel graph for assessment of potential publication bias in studies of TAM density in patients with breast cancer Publication bias of OS **A**., DFS **B**., RFS **C**., BCSS **D**. of the meta-analysis showed no statistical significance (*p* > 0.05) using Begg's test.

We use funnel plot analysis and Egger'/Begg' test to evaluate publication bias and there was no statistical difference for OS, DFS, RFS and BCSS with P value of 0.574/ 0.787, 0.019 /0.293, 0.368/ 0.805, 0.314/ 0.322 separately. Begg's funnel plots with pseudo 95% confidence limits of the four groups were listed in Figure 6A-6D.

### Sensitivity analysis

**Figure 7:**
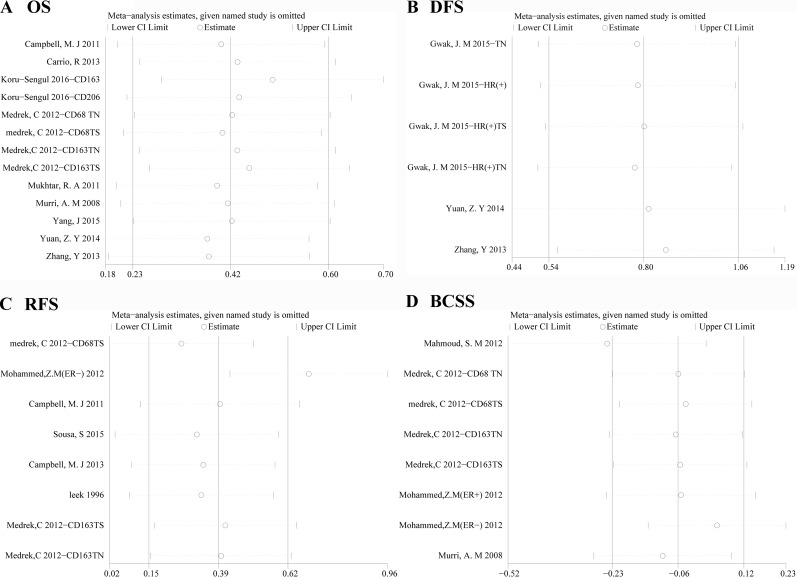
Sensitivity for included studies The effect of single study was evaluated on the whole results of OS **A**., DFS **B**., RFS **C**., BCSS **D**. in this meta-analysis.

Results of removal of each study at a time could be seen in Figure 7A-7D. For the OS and DFS analysis, removal of each study didn't change HR significantly. For the RFS analysis, removal of “Mohammed,Z.M(ER-) 2012” had an important effect of the results. For BCSS, removal of “Mahmoud, SM 2012” also affect the overall results.

## DISCUSSION

Mountains of studies have illustrated the role of TAMs in cancer initiation and progression [2, 4, 22–24]. But the pertinence between TAMs and outcomes of breast cancer hasn't been illuminated. In our meta-analysis, we chose OS, DFS, RFS and BCSS to assess risk of high macrophage density. Moreover, we analyzed the correlation between clinicopathological features and TAMs infiltration and found high TAMs infiltration was related to both poor survival rates and malignant biological behavior.

Our meta-analysis of 16 studies with 4541 patients in total shown that high density of TAMs was related to worse OS and DFS. What's more, we found that the prognostic significance of TAMs was affected by different biomarker, TAMs location and ER receptor status. High density of TAMs was related to poor prognosis and subgroup analysis showed that CD68 was better than M2 specific marker CD163 and CD206 alone in predicting OS. And results of subgroup analysis of location shown both TN+TS and TS group signified worse OS, while there was no difference of TAMs in TN group. All the results indicated that CD68 and TAMs in tumor stroma were strongly associated with worse OS. For DFS, high density of TAMs had the similar prognostic significance as OS outcome.

Results of elevated TAMs of RFS shown no obvious correlation. And the sensitivity analysis shown the exclusion of study of “Mohammed,Z.M(ER-) 2012” obviously affect the association between TAMs density and RFS. This article mentioned above reported an improved recurrence-free survival of CD68 TAMs in ER- breast cancer, which was opposite to some other studies [25–27]. With regard to BCSS, results showed no statistical significance, HR = 0.95(0.79,1.13). So far, the reasons for such discrepancies using similar methodology are unclear, however, some evidence declares macrophage markers, such as CD68 may be expressed by other non-myeloid tissues in cancer [28]. Besides, the heterogeneity of RFS and BCSS is considerable, maybe the results should be considered cautiously.

Furthermore, we analyzed association between TAMs infiltration and several clinicopathological parameters. Results indicated high density of TAMs was related to younger age, larger size, high histologic grade, negative hormone receptor status, malignant phenotype and vascular invasion, which were all crucial factors for predicting the prognosis. Thus, high density of TAMs may lead to poor survival rates by promoting tumor proliferation, migration and invasion.

This study has some important implications in breast cancer. Firstly, it demonstrates high TAMs are related to worse outcome, which indicates that TAMs may be a potential therapeutic target. Secondly, it shows the tissue distribution and CD68 biomarker of TAMs has an important role in prognostic prediction.

There are also limitations in this meta-analysis. First of all, the markers and cut-off values for assessing TAMs expression of individual studies are inconsistent and cut-off value may be a source of considerable interstudy heterogeneity. Besides, although Begg's and Egger's test were performed and there was no statistical significance. Results should be interpreted cautiously because we only include studies with available HR value or K-M survival curves with necessary data.

In short, our analyses show that high density of TAMs in breast cancer tissues, especially CD68+ TAMs in tumor stroma, is associated with worse prognosis in human breast cancer. As main kind infiltrating cells in tumor microenvironment, TAMs have a close relationship with tumor cells by direct or indirect function [29]. Some factors secreted by TAMs also been reported to be correlated with poor prognosis [30, 31]. Targeted therapies directly targeting TAMs, or reprogramming TAMs to M1phenotype could be promising in improving survival rates of breast cancer patients.

## MATERIALS AND METHODS

### Search strategy

This meta-analysis was conducted according to PRISMA guidelines. We searched PubMed (MEDLINE), Web of science and Embase from their inception until July 1, 2016 and the records and results are shown in Figure 1. The following Mesh Terms or key words were used in the search: “cancer”, “tumor”, “neoplasm”, “carcinoma”, “macrophages” and “breast cancer”. The language was restricted to English. The references from identified articles were manually searched for additional relevant records.

### Inclusion criteria

Inclusion criteria for primary studies were as follows:(1) proven diagnosis of breast cancer by pathology ; (2) without previous cancer history; (3) evaluating TAMs by CD68, CD206, CD163 using immunochemistry; (4) reporting the correlation of TAMs with OS, DFS, RFS, BCSS and clinicopathological features; (5) full-text studies published in English. Only articles conforming to all the five conditions above were selected, or they would be excluded. Two authors (ZXX and QJK) independently extracted all data. If there were disagreements, we solved by reaching a consensus or discussion with a third investigator. The names of authors and the medical centers involved were examined in each publication to avoid repeated data. The quality of included studies was assessed by using the Newcastle-Ottawa Scale checklist (NOS).

### Statistical analyses

Included studies were divided into four groups for analysis: OS, DFS, RFS and BCSS. To integrate survival results, data of TAMs density of survival in individual study was extracted by estimating HRs and 95% confidence interval values. Firstly, we searched original article to get HRs and 95% CI. If data were only available in the form of figures, we read Kaplan-Meier curves by Engauge Digitizer version 4.1 (free software downloaded from http://sourceforge.net) and extracted survival data HRs and 95%CI [32]. Moreover, data of clinicopathological features was extracted in study available by estimating ORs. The heterogeneity of included studies across the results was assessed by using I2 statistics and P value, and if I^2^ > 50% or *P* < 0.1, the results was considered statistically significant and random-effects models are employed. If *P*≥0.1 and I^2^ ≤ 50%, fixed-effects models are employed. If the 95% CI didn't overlap 1, or *P* < 0.05, results would be considered statistically significant. Statistical analyses were performed using Stata 13.0 (Stata Corporation, College Station, TX).

### Sensitivity analyses

We carried out sensitivity analysis, also named influence analysis, to evaluate the effect of single study on the whole results and meanwhile try to find the origin of heterogeneity for each survival outcome group.

### Publication bias

Publication bias was assessed graphically using funnel plots, and funnel plot Symmetry was evaluated by Begg's and Egger's linear regression method (*p* < 0.05 was considered statistically significant publication bias).
